# Detection of Aryl Hydrocarbon Receptor Activation by Some Chemicals in Food Using a Reporter Gene Assay

**DOI:** 10.3390/foods5010015

**Published:** 2016-02-25

**Authors:** Yoshiaki Amakura, Tomoaki Tsutsumi, Morio Yoshimura, Masafumi Nakamura, Hiroshi Handa, Rieko Matsuda, Reiko Teshima, Takahiro Watanabe

**Affiliations:** 1 College of Pharmaceutical Sciences, Matsuyama University, Matsuyama, Ehime 790-8578, Japan; myoshimu@cc.matsuyama-u.ac.jp; 2 Division of Foods, National Institute of Health Sciences, Setagaya-ku, Tokyo 158-8501, Japan; tutumi@nihs.go.jp (T.T.); matsuda@nihs.go.jp (R.M.); rteshima@nihs.go.jp (R.T.); tawata@nihs.go.jp (T.W.); 3 Hiyoshi Corporation, Omihachiman, Shiga 523-8555, Japan; m.nakamura@hiyoshi-es.co.jp (M.N.); handa@hiyoshi-es.co.jp (H.H.)

**Keywords:** aryl hydrocarbon receptor, reporter gene assay, food hygiene, polycyclic aromatic hydrocarbon, pesticide, amino acid

## Abstract

The purpose of this study was to examine whether a simple bioassay used for the detection of dioxins (DXNs) could be applied to detect trace amounts of harmful DXN-like substances in food products. To identify substances with possible DXN-like activity, we assessed the ability of various compounds in the environment to bind the aryl hydrocarbon receptor (AhR) that binds specifically to DXNs. The compounds tested included 19 polycyclic aromatic hydrocarbons (PAHs), 20 PAH derivatives (nitrated, halogenated, and aminated derivatives), 23 pesticides, six amino acids, and eight amino acid metabolites. The AhR binding activities (AhR activity) of these compounds were measured using the chemical activated luciferase gene expression (CALUX) reporter gene assay system. The majority of the PAHs exhibited marked AhR activity that increased in a concentration-dependent manner. Furthermore, there was a positive link between AhR activity and the number of aromatic rings in the PAH derivatives. Conversely, there appeared to be a negative correlation between AhR activity and the number of chlorine residues present on halogenated PAH derivatives. However, there was no correlation between AhR activity and the number and position of substituents among nitrated and aminated derivatives. Among the pesticides tested, the indole-type compounds carbendazim and thiabendazole showed high levels of activity. Similarly, the indole compound tryptamine was the only amino acid metabolite to induce AhR activity. The results are useful in understanding the identification and characterization of AhR ligands in the CALUX assay.

## 1. Introduction

Food products can be contaminated by a number of harmful substances, including dioxins (DXNs) and polycyclic aromatic hydrocarbons (PAHs); previous studies have focused on evaluating the health risks associated with these substances as well as ways to manage these health risks [1,2]. Human exposure to DXNs mainly occurs through foods, such as fish, meat, and dairy products. PAHs are formed mainly during cooking and during processes, such as smoking. There are currently 16 PAHs that are considered worthy of health concern by the EU (EU priority PAHs); the EU has set maximum limits of PAHs in foodstuffs. Additionally, some pesticides can be detected at relatively high rates in foodstuffs. These findings prompted us to investigate the aryl hydrocarbon receptor (AhR) activities of DXNs, PAHs, and pesticides. Although trace amounts of these contaminants pose a low health risk, the risk management of the maximum limits of their residual levels is crucial to food safety.

The DXN and PAH families are comprised of various analogs that have similar chemical structures. Detection and quantification of these compounds in food products are currently achieved using a GC-MS approach, wherein the levels of DXNs and PAHs are determined using standardized methods as indicators [3,4]. However, because this approach is both complicated and costly, various simplified analytical methods have been proposed. For example, bioassays using AhR are commonly performed to measure levels of environmental pollutants, including DXNs [5]. Indeed, these assays led to the discovery that AhR is a dioxin receptor. It has, therefore, been suggested that AhR binding could be associated with the biological toxicity of these pollutants. In addition, AhR was reported to be activated by binding of PAHs [6]. The AhR-binding properties of PAHs could enable bioassay screening of foodstuffs for both DXNs and PAHs. Furthermore, because AhR bioassays are rapid and inexpensive, they could provide a convenient alternative to the current screening methods for toxic compounds, as well as other aforementioned substances.

To assess the efficacy of such an assay for detecting food contaminants, we developed a simple AhR-based bioassay technique to detect potentially hazardous substances with DXN-like activity, and determined whether there was a correlation between chemical structure and AhR activity. For this assay, AhR activity was evaluated using the chemical activated luciferase gene expression (CALUX) system, which was originally designed to detect DXNs [7]. The CALUX assay has been widely used for screening for dioxins in food and feed [8,9]. It is important to know the dioxin-like activity for various compounds in food to understand the possible influence of these compounds on our screening assay. The compounds examined included 39 PAHs (including nitrated derivatives, halogenated derivatives, and aminated derivatives), 23 pesticides, and 14 amino acids or amino acid metabolites.

## 2. Experimental Section

### 2.1. Chemicals and Reagents

**Figure 1:**
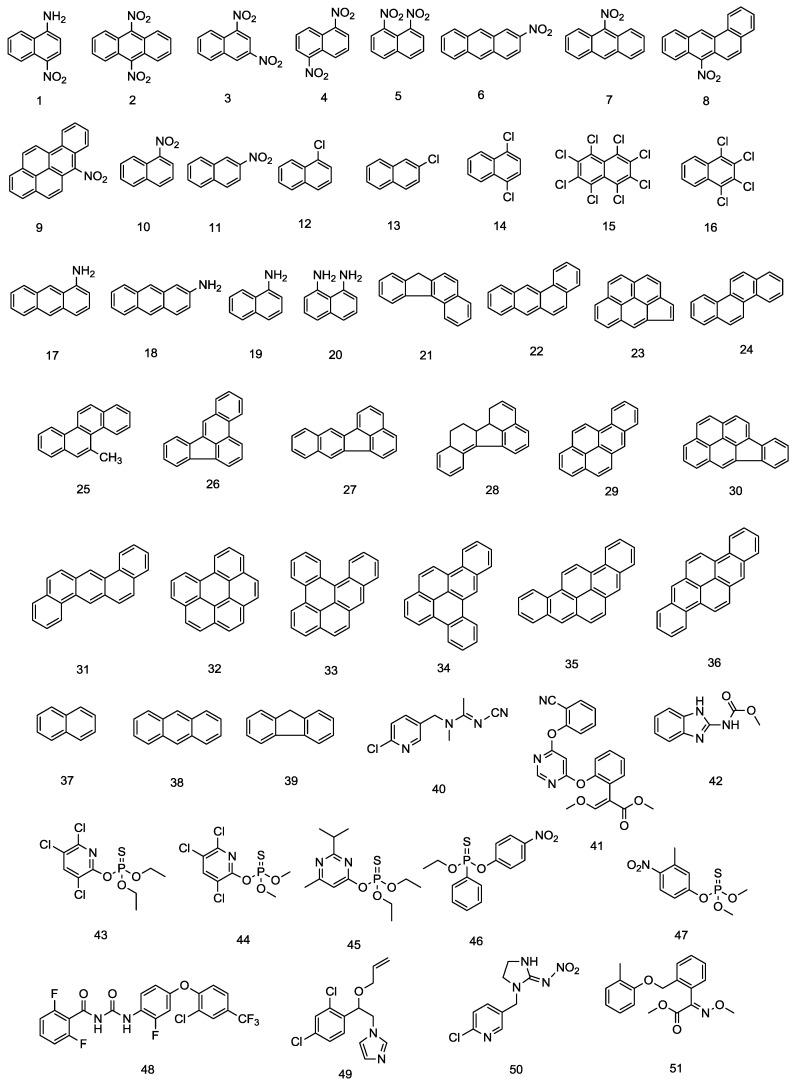

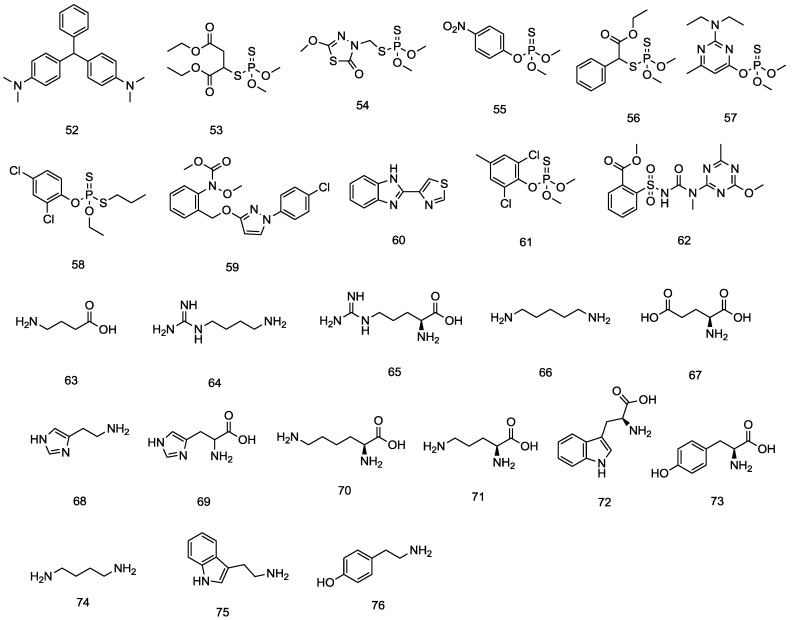
Chemical structures of the compounds tested.

The following 39 PAHs (including nitrated, halogenated, and aminated derivatives) were used in this study: benzo(*c*)fluorene, 1,2-benzanthracene (benzo(*a*)anthracene), cyclopenta(*c,d*)pyrene, chrysene, 5-methylchrysene, benzo(*b*)fluoranthene, benzo(*k*)fluoranthene, benzo(*j*)fluoranthene, benzo(*a*)pyrene, indeno(1,2,3-*c,d*)pyrene, dibenzo(*a,h*)anthracene, benzo(*g,h,i*)perylene, dibenzo(*a,l*)pyrene, dibenzo(*a,e*)pyrene, dibenzo(*a,i*)pyrene, dibenzo(*a,h*)pyrene, 1-amino-4-nitronaphthalene, 9,10-dinitroanthracene, 1,3-dinitronapthalene, 1,5-dinitronaphthalene, 1,8-dinitronaphtalene, 2-nitroanthracene, 9-nitroanthracene, 7-nitrobenzo(*a*)anthracene, 6-nitrobenzo(*a*)pyrene, 1-nitronaphthalene, 2-nitronaphthalene, 1-chloronaphthalene, 2-chloronaphthalene, 1,4-dichloronaphthalene, octachloronaphthalene, 1,2,3,4-tetrachloronaphthalene, 1-aminoanthracene, 2-aminoanthracene, 1-aminonaphthalene, 1,8-diaminonaphthalene, naphthalene, anthracene, and fluorene were purchased from Kanto Chemical (Tokyo, Japan). The following 23 pesticide residues were also used: malathion, chlorpyrifos, diazinon, prothiofos, pirimiphos-methyl, fenitrothion, ethyl-*p*-nitrophenyl phenylthiophosphonothiate (EPN), tolclofos-methyl, parathion-methyl, phenthoate, chlorpyrifos-methyl, methidathion, imazalil, carbendazim, leucomalachite green, imidacloprid, acetamiprid, thiabendazole, azoxystrobin, tribenuron-methyl, flufenoxuron, pyraclostrobin, and kresoxim-methyl (Kanto Chemical). The amino acids l-tryptophan, l-glutamic acid, l-tyrosine, l-lysine, l-arginine, and histidine, as well as the amino acid metabolites tryptamine, 4-aminobutanoic acid, tyramine, putrescine, cadaverine, and histamine were purchased from Wako Pure Chemical Industries (Osaka, Japan). l-Ornithine and agmatine were obtained from Tokyo Chemical Industry (Tokyo, Japan) (Table 1, Figure 1). 2,3,7,8-Tetrachlorodibenzo-*p*-dioxin (TCDD) and dimethyl sulfoxide (DMSO) were purchased from Wako Pure Chemical Industries. All chemicals were of analytical grade.

### 2.2. Evaluation of AhR Activity

AhR activation mediated by target compounds was evaluated using the CALUX assay. The CALUX assay was conducted as follows: mouse hepatoma H1L6.1c2 cells (*ca*. 1.5 × 10^5^ cells/well) [10] were cultured in 96-well culture plates, and the target compounds were dissolved in DMSO and added at final concentrations of 0.1–100,000 nM in 4–6 steps. The final DMSO concentration was 1% in the cell culture medium. The plates were then incubated at 37 °C in 5% CO_2_ for 24 h for optimal expression of luciferase activity. After incubation, cell viability was confirmed by microscopy, the medium was removed, and the cells were lysed. Luciferin was then added as a substrate, and luciferase activity was determined using a luminometer (Centro LB 960; Berthold Technologies, Bad Wildbad, Germany) and recorded as relative light units (RLUs). Results are presented as mean ± SD of at least two or three independent measurements per experiment.

## 3. Results and Discussion

**Figure 2:**
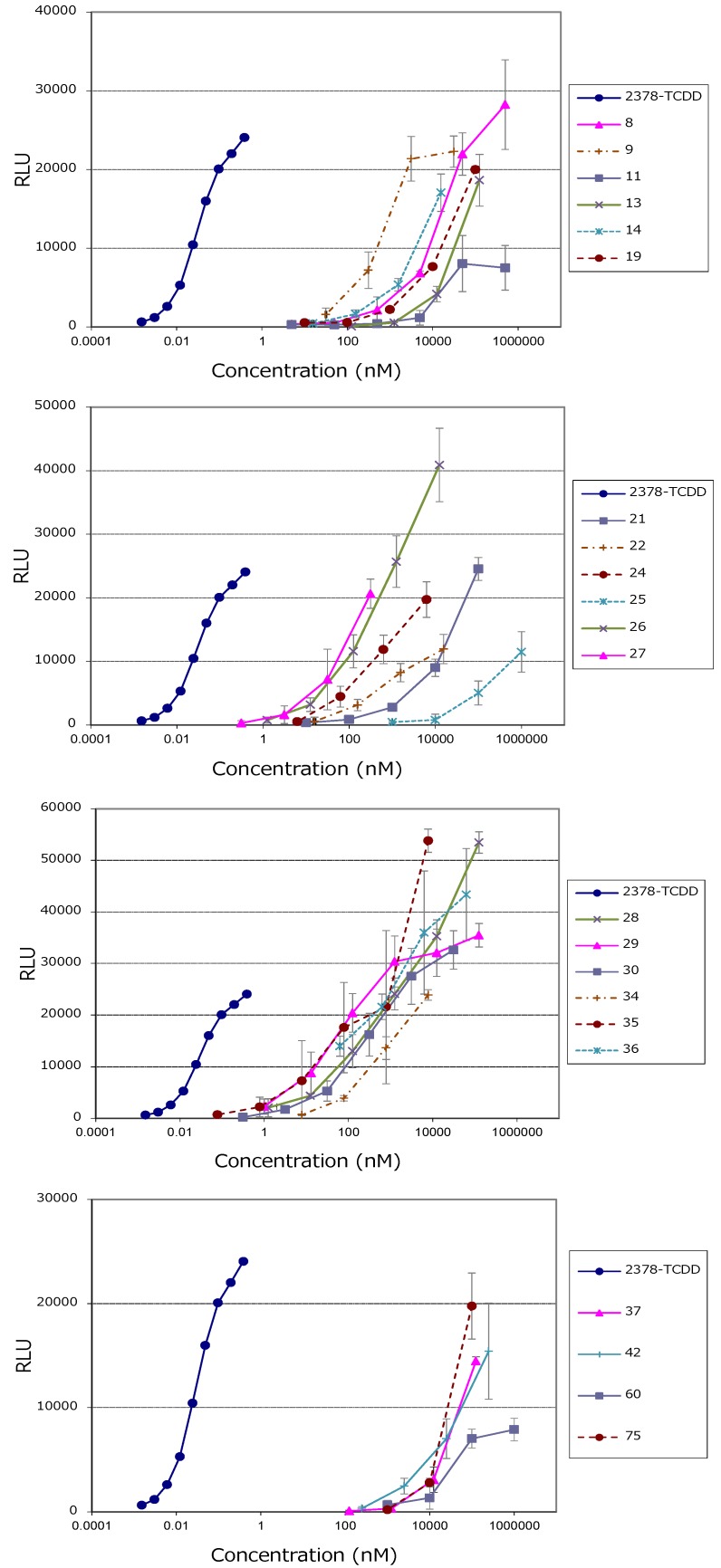
Concentration-response curve of luciferase activity induced by the selected compounds and 2,3,7,8-Tetrachlorodibenzo-*p*-dioxin (TCDD). Approximately 1.5 × 10^5^ cells/well were cultured in 96-well culture plates and exposed to 0.1–100,000 nM concentrations of the selected compounds for 24 h. Cells were then lysed, and luciferase activity was measured using luciferin. The numbers within the graph correspond to the numbers in Table 1.

The compounds examined in this study are listed in Table 1, and their chemical structures are shown in Figure 1. In addition, the results for compounds that showed AhR activity are depicted in Figure 2 and Table 2.

### 3.1. PAHs

Of the 19 PAH compounds examined, 13 induced marked AhR activity in the CALUX assay. Furthermore, these PAH compounds increased AhR activity in a concentration-dependent manner (Figure 2 and Table 2). The only PAHs that failed to induce activity were cyclopenta(*c,d*)pyrene (**23**), dibenzo(*a,h*)anthracene (**31**), benzo(*g,h,i*)perylene (**32**), dibenzo(*a,l*)pyrene (**33**) and anthracene (**39**); while fluorene (**38**) induced only weak activity. Of the 20 PAH derivatives (nitrated, halogenated and aminated PAHs), 7-nitrobenzo(*a*)anthracene (**8**), 6-nitrobenzo(*a*)pyrene (**9**), 2-chloronaphthalene (**13**), 1,4-dichloronaphthalene (**14**) and 1-aminonaphthalene (**19**) exhibited strong activity, while 2-nitroanthracene (**11**) and 2-aminoanthracene (**18**) exhibited weak activity (Figure 2 and Table 2). According to scientific reports on the carcinogenicity of PAHs, dibenzo(*a,i*)pyrene (**35**) is classified as a group 2B carcinogen (possibly carcinogenic to humans), while benzo(*g,h,i*)perylene (**32**) is listed as a group 3 carcinogen (unclassifiable in regards to carcinogenicity to humans) [11]. Although these discrepancies indicate no correlation between carcinogenicity and AhR activity, further studies will be necessary to determine whether carcinogenicity is associated with the degree of toxicity of PAHs. In recent years, concerns have been raised regarding the possible presence of PAH derivatives in foods [12]. Our results indicate a potential positive correlation between the quantities of aromatic rings in the associated PAH derivatives and AhR activity: as the number of aromatic rings increased, AhR activity tended to increase. Conversely, there appeared to be a negative correlation between the number of chlorine atoms associated with halogenated PAH derivatives (*i.e.*, chlorinated compounds) and AhR activity. Meanwhile, differences in the number and positions of nitro groups associated with nitrated derivatives did not affect the intensity of AhR activity; however, increased activity was observed for compounds containing four or more aromatic rings. 1-Aminonaphtalene (**19**) was the only aminated derivative to induce AhR activity, and no correlation between the structure and activity was observed (Figure 1 and Figure 2, and Table 2).

As shown in Figure 2, the sensitivity of this assay to PAHs was approximately 1000 times weaker than that to TCDD; however, the values of the maximum RLUs were approximately two times greater. This may have been due to the differences between DXNs and PAHs in regards to metabolic rates and the genes involved. It is also possible that this is due to cross-talk between the AhR and compounds associated with cell signaling and protein degradation pathways [13]. Nevertheless, our findings suggest that this assay could be applied to the analysis of PAH levels in foods, and may therefore provide a more cost-effective test for use in the food industry in the future.

A few studies have examined AhR activities of PAHs using stable rat H4IIE hepatoma cells transfected with AhR-controlled luciferase reporter gene plasmid after 24-hour incubation [14,15]. Our results for the relative potencies (REP) for the eleven PAHs (**22, 24–30, 34–36**) as shown in Table 2, when compared with the previously reported REP values based on EC_20_ or EC_25_ [14,15], showed significant differences only for 5-methylchrysene (**25**), while all the other PAHs were different by at most about one order of magnitude. Metabolic differences between mouse and rat cell lines for the PAHs may account for the differences.

### 3.2. Pesticides

Examination of 23 pesticides revealed that carbendazim (**42**) and thiabendazole (**60**), both of which have an indole skeleton (Figure 1), exhibited strong AhR activation (Figure 2 and Table 2). These results were consistent with those of previous studies that found that indole-type compounds exhibit AhR activity [16,17]. Takeuchi *et al.* [18] examined AhR activity of fifteen pesticides (**42–47, 49, 50, 53–56, 58, 60, 61**) tested in the present study using the recombinant mouse hepatoma cell line (DR-EcoScreen cells) after 24-hour incubation. There was good comparability with both results excepting chlorpyrifos (**43**). They found chlorpyrifos (**43**) has AhR activity, although it had no AhR activity in our CALUX assay. The difference in response to chlorpyrifos (**43**) is unclear at this time but may be related to the structure of the dioxin-responsive luciferase reporter plasmids to generate stable cell lines.

### 3.3. Amino Acids and Their Metabolites

Among the 14 amino acids or amino acid metabolites examined to study of the relationship between structure and AhR activity, AhR activity was only induced by tryptamine (**75**). Similar to carbendazim and thiabendazole, tryptamine has an indole skeleton (Figure 1 and Figure 2, and Table 2). Health-Pagliuso, *et al.* [19] reported that l-tryptophan (**72**) and its metabolite, tryptamine (**75**) induced AhR-dependent gene expression using the recombinant mouse hepatoma cell line (H1L1.1c2) after 4-hour incubation. However, we observed that l-tryptophan (**72**) did not show any agonistic activity at the tested concentrations. This is probably due to the different characteristics in the recombinant mouse hepatoma cell lines. The H1L1.1c2 cell allows for detection of AhR-active compounds that generally inactivated by metabolism in longer-time incubations.

A variety of genes, such as the cytochrome P4501A1 gene, is shown to be directly regulated by the AhR system in the CALUX cell line. Therefore, the CALUX assay is considered more appropriate for detecting AhR-active compounds that are relatively stable metabolically during the exposure time, and less appreciate for detecting AhR-active compounds that are metabolically converted to non-AhR binding forms in shorter-time incubations. Metabolically labile compounds, (*i.e.*, PAHs) tend to exhibit low AhR activities in the CALUX assay.

It is not recommended to conclude that the toxicities of chemicals should only be determined by their AhR activation. Although AhR activation is the key in causing dioxin-like toxicities, AhR activation followed by various signal activation expressions are also important in causing toxicity. Our results simply show if the tested compounds activated the AhR. Our results are useful however, in understanding the identification and characterization of AhR ligands in the CALUX assay.

## 4. Conclusions

To identify toxic substances with DXN-like activity and to examine whether there was a correlation between AhR activities and the chemical structures of the candidate substances, we assessed the AhR activities of 39 PAHs (including nitrated derivatives, halogenated derivatives, and aminated derivatives), 23 pesticides, six amino acids, and eight amino acid metabolites using the CALUX assay. The majority of the PAHs examined (**13** of **19**) exhibited marked AhR activity. Notable, those PAH derivatives that contained multiple aromatic rings tended to be associated with greater activity. Conversely, lower levels of AhR activity were associated with halogenated derivatives that had multiple chlorine atoms. Meanwhile, there was no correlation between AhR activity and the number and position of nitrate and amine groups on the nitrated and aminated derivatives. Of the pesticides, amino acids, and amino acid metabolites tested, only those compounds with indole structures exhibited marked AhR activity.

In this study, we identified numerous AhR active (DXN-like) substances using the convenient CALUX assay for AhR ligand activity. Although the amounts of these compounds found in food would likely be minute, further studies are needed to assess the effects of these compounds on human health and to enhance the safety management of ordinary foods.

## Figures and Tables

**Table 1:** List of the compounds tested.

No.	PAHs	No.	Pesticides Residues
1	1-Amino-4-nitronaphthalene	40	Acetamiprid
2	9,10-Dinitroanthracene	41	Azoxystorobin
3	1,3-Dinitronaphthalene	42	Carbendazim
4	1,5-Dinitronaphthalene	43	Chlorpyrifos
5	1,8-Dinitronaphthalene	44	Chlorpyrifos methyl
6	2-Nitroanthracene	45	Diazinon
7	9-Nitroanthracene	46	EPN
8	7-Nitrobenzo(*a*)anthracene	47	Fenitrothion
9	6-Nitrobenzo(*a*)pyrene	48	Flufenoxuron
10	1-Nitronaphthalene	49	Imazalil
11	2-Nitroanthracene	50	Imidacloprid
12	1-Chloronaphthalene	51	Kresoxim methyl
13	2-Chloronaphthalene	52	Leucomalachite green
14	1,4-Dichloronaphthalene	53	Malathion
15	Octachloronaphthalene	54	Methidathion
16	1,2,3,4-Tetrachloronaphthalene	55	Parathion methyl
17	1-Aminoanthracene	56	Phenthoate
18	2-Aminoanthracene	57	Primiphos methyl
19	1-Aminonaphthalene	58	Prothiofos
20	1,8-Diaminonaphthalene	59	Pyraclostrobin
21	Benzo(*c*)fluorene	60	Thiabendazole
22	Benzo(*a*)anthracene	61	Tolclofos methyl
23	Cyclopenta(*c,d*)pyrene	62	Tribenuron methyl
24	Chrysene	**No.**	**Amino acids and their metabolites**
25	5-Methylchrysene	63	4-Aminobutanoic acid
26	Benzo(*b*)fluoranthene	64	Agmatine
27	Benzo(*k*)fluoranthene	65	l-Arginine
28	Benzo(*j*)fluoranthene	66	Cadaverine
29	Benzo(*a*)pyrene	67	l-Glutamic acid
30	Indeno(1,2,3-*c,d*)pyrene	68	Histamine
31	Dibenzo(*a,h*)anthrathene	69	Histidine
32	Benzo(*g,h,i*)perylene	70	l-Lysine
33	Dibenzo(*a,l*)pyrene	71	l-Ornithine
34	Dibenzo(*a,e*)pyrene	72	l-Tryptophan
35	Dibenzo(*a,i*)pyrene	73	l-Tyrosine
36	Dibenzo(*a,h*)pyrene	74	Putrescine
37	Naphthalene	75	Tryptamine
38	Fluorene	76	Tyramine
39	Anthracene		

**Table 2:** Responses of the reporter gene system to the selected compounds.

Compounds	EC _RLU5000_^a^, nM (REP) ^b^
TCDD	0.01 (1)
7-Nitrobenzo(*a*)anthracene (**8**)	1.70 × 10^3^ (5.9 × 10^−6^)
6-Nitrobenzo(*a*)pyrene (**9**)	1.76 × 10^2^ (5.7 × 10^−5^)
2-Nitroanthracene (**11**)	3.81 × 10^4^ (2.6 × 10^−7^)
2-Chloronaphthalene (**13**)	2.56 × 10^4^ (3.9 × 10^−7^)
1,4-Dichloronaphthalene (**14**)	1.46 × 10^3^ (6.8 × 10^−6^)
1-Aminonaphthalene (**19**)	5.00 × 10^3^ (2.0 × 10^−6^)
Benzo(*c*)fluorene (**21**)	2.31 × 10^3^ (4.3 × 10^−6^)
Benzo(*a*)anthracene (**22**)	8.87 × 10^2^ (1.1 × 10^−5^)
Chrysene (**24**)	2.41 × 10^2^ (4.1 × 10^−5^)
5-Methylchrysene (**25**)	2.97 × 10^4^ (3.4 × 10^−7^)
Benzo(*b*)fluoranthene (**26**)	19.7 (5.1 × 10^−4^)
Benzo(*k*)fluoranthene (**27**)	2.36 (4.2 × 10^−3^)
Benzo(*j*)fluoranthene (**28**)	18.8 (5.3 × 10^−4^)
Benzo(*a*)pyrene (**29**)	3.72 (2.7 × 10^−3^)
Indeno(1,2,3-*c*,*d*)pyrene (**30**)	20.3 (4.9 × 10^−4^)
Dibenzo(*a*,*e*)pyrene (**34**)	1.13 × 10^2^ (8.8 × 10^−5^)
Dibenzo(*a*,*i*)pyrene (**35**)	6.17 (1.6 × 10^−3^)
Dibenzo(*a*,*h*)pyrene (**36**)	9.32 (1.1 × 10^−3^)
Naphthalene (**37**)	3.77 × 10^4^ (2.7 × 10^−7^)
Carbendazim (**42**)	1.30 × 10^4^ (7.7 × 10^−7^)
Thiabendazole (**60**)	7.10 × 10^4^ (1.4 × 10^−7^)
Tryptamine (**75**)	2.51 × 10^4^ (4.0 × 10^−7^)

^a^ Concentration of compounds required to produce a luciferase activity of 5000 relative light unit (RLU). Values were calculated from the slope of the linear portion of each dose-response curve. ^b^ REPs were determined as the ratios of the concentrations of TCDD and the tested compound at EC _RLU5000_.
